# Pharmaceutical Insights Into Ammi and Parsley: Evaluating Antioxidant Activity, Total Phenolic Content, and Kidney Stone Disintegration Properties

**DOI:** 10.1155/adpp/5522905

**Published:** 2025-02-20

**Authors:** Ruba Malkawi, Khairat Battah, Mohammad Alkhreisat

**Affiliations:** ^1^Department of Pharmacy, Jadara University, P.O. Box 733, Irbid 21110, Jordan; ^2^Department of Pathology, Microbiology and Forensic Medicine, Faculty of Medicine, Al-Balqa Applied University, Al Salt 19117, Jordan; ^3^Department of Special Surgery, Faculty of Medicine, Al-Balqa Applied University, Al Salt 19117, Jordan

**Keywords:** *Ammi visnaga*, antioxidant activity, *Petroselinum crispum*, pharmaceutical investigation, total phenolic content

## Abstract

This study investigated the pharmaceutical potential of extracts from *Ammi visnaga* (Ammi) and *Petroselinum crispum* (Parsley), specifically focusing on their antioxidant activity, total phenolic content, and efficacy in disintegrating calcium oxalate kidney stones. Ammi and Parsley extracts, known for their traditional medicinal uses, contain bioactive compounds with significant antioxidant properties that have attracted attention in pharmaceutical research. Oxidative stress, a key factor in various physiological disorders, underscores the importance of antioxidants in the mitigation of cellular damage. Our investigation revealed concentration-dependent enhancements in antioxidant activity and total phenolic content in both Ammi and Parsley extracts, indicating their potential as natural antioxidant agents. Furthermore, both extracts were effective in reducing the size of calcium oxalate stones, with the Ammi extract demonstrating superior stone-disintegration properties. Dissolution studies have provided valuable insights into the release kinetics of phenolic compounds and antioxidant activity, suggesting sustained therapeutic potential. Overall, Ammi and Parsley extracts show promise in pharmaceutical development, offering alternative therapeutic avenues for managing oxidative stress-related conditions and kidney stone formation.

## 1. Introduction

The utilization of natural products for medicinal purposes has garnered significant attention owing to their potential therapeutic benefits and minimal adverse effects compared with synthetic alternatives [1]. Among these, *Ammi visnaga* (Ammi) and *Petroselinum crispum* (Parsley) have long been recognized in traditional medicine because of their diverse pharmacological properties. Ammi, commonly known as toothpick weed, and Parsley, a culinary herb, possess bioactive constituents that exhibit antioxidant properties and may hold promise in managing various health conditions. The production of reactive oxygen species (ROS), characterized by a single unpaired electron, induces oxidative stress and is a pivotal factor in the development of various physiological ailments. These include cellular damage; aging; cancer; and hepatic, neurodegenerative, cardiovascular, and renal disorders [2]. *Ammi visnaga*, a plant native to the Mediterranean region, contains compounds such as khellin and visnagin, known for their vasodilatory action and potential in treating conditions such as hypertension and angina [3] and is very useful for renal stone deposits [4]. Parsley, which is rich in flavonoids, carotenoids, and essential oils, has been historically used for its diuretic, anti-inflammatory, and antimicrobial properties [5]. These plants have garnered scientific interest because of their antioxidant potential, which is attributed to the presence of phenolic compounds that play a crucial role in neutralizing free radicals and mitigating oxidative stress-induced damage in the body [6]. Antioxidants obtained from natural sources, such as Ammi and Parsley, have shown promise in fighting oxidative stress, an important contributor to several chronic diseases, including cardiovascular disorders, cancer, and neurodegenerative conditions [2]. The total phenolic content of these plants serves as a marker for their antioxidant potential, with higher phenolic concentrations correlating with increased scavenging activity against ROS, thus conferring greater health benefits [7]. ROS, unique unpaired electron-ion, generation, has an important contribution in oxidative stress induction and as a key step in many pathogenesis. Oxidative stress interferes with cellular homeostasis and gives rise to diseases such as aging, cancer, liver, cardiovascular, nervous systems, and kidney disorders. The function of ROS in these diseases has been investigated in a wide range. For example, Gallie et al. [8] demonstrated the important relationship between oxidative stress and cellular injury and focus on how a dissociation in production and antioxidant defense mechanisms leads to disease accumulation. Moreover, evidence has shown the protective role of natural antioxidants in preventing ROS-mediated damage. Phytochemical components obtained from plants such as *Ammi visnaga* and *Petroselinum crispum*, containing phenolic compounds, have demonstrated high antioxidant properties with the ability of scavenging free radicals and attenuating oxidative stress. These results highlight the need for further investigation of plant sources of antioxidants as treatment options for oxidative stress-associated disorders.

Pharmaceutical studies exploring the dissolution and absorption profiles of Ammi and Parsley extracts further underscore their therapeutic relevance. Dissolution studies, specifically in simulated gastric and intestinal conditions, provide valuable insights into the bioavailability and efficacy of plant-derived compounds [9]. Remarkably, our research has shown superior dissolution of Ammi in the intestinal environment, highlighting its potential for enhanced absorption, whereas Parsley exhibited optimal dissolution in the stomach, suggesting targeted delivery and action.

**Figure 1:**
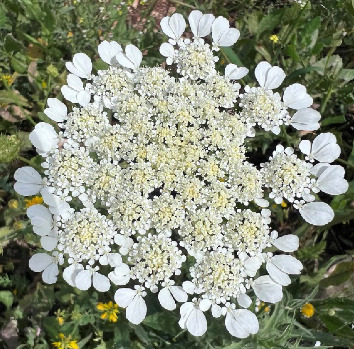
*Ammi visnaga* (Ammi).

**Figure 2:**
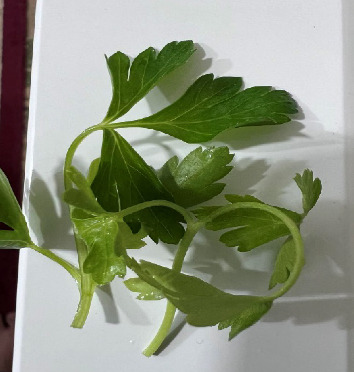
*Petroselinum crispum* (Parsley).

In this study, we investigated the therapeutic potential of Ammi and Parsley extracts (Figures 1 and 2) in disintegrating oxalate kidney stones, a common urinary tract ailment. The grounded forms of Ammi and Parsley were subjected to aqueous extraction, yielding the concentrated extracts used for experimentation. Notably, soaking oxalate stones in variable concentrations of these extracts resulted in a significant reduction in stone size, particularly in the Ammi extract, indicating its potent stone-disintegrating properties.

In addition, we evaluated the total phenolic content and antioxidant activity of Ammi and Parsley extracts at different concentrations. Specifically, we also assessed Ammi and Parsley extracts total phenolic content and antioxidant activity at various concentrations. For a more complete picture of how these assays have been used to investigate the health targetability advantages of plants, such studies from the literature should be considered. For instance, the research by Abualzulof [10] described the role of the maturity stage in polyphenolic composition and bioactivity of *Ficus rubiginosa* extracts and found that phenolic content and corresponding antioxidant activities depended considerably and changes in the development stage of plant. This confirms the role of polyphenolic compounds as sources of antioxidant activities, an issue further discussed in the present investigation. These comparative observations not only confirm the usage of the developed methods but also shed light on the wider applicability of antioxidant and phenolic assays in assessing the therapeutic potential of medicinal plants [11].

Our results revealed concentration-dependent increases in both the total phenolic content and antioxidant inhibition percentage, underscoring the dose-dependent antioxidant efficacy of these botanicals. This study emphasizes the therapeutic potential of Ammi and Parsley extracts in managing oxidative stress-related conditions and disintegrating oxalate kidney stones [6, 12, 13]. Their significant antioxidant activity, coupled with their high phenolic content and positive dissolution profiles, make them promising candidates for further pharmaceutical development and clinical exploration in the realm of natural medicine.

## 2. Materials and Methods

### 2.1. Reagents and Chemicals

All the solvents used were of HPLC grade. Folin–Ciocalteu reagent, calcium oxalate, and gallic acid 2,2- were purchased from Sigma-Aldrich Chemical, while diphenyl-l-picrylhydrazyl (DPPH•) was obtained from POCH (Poland).

Note: Gallic acid is a naturally occurring organic compound that belongs to the class of phenolic acids. Its chemical name is 3,4,5-trihydroxybenzoic acid, and it is characterized by three hydroxyl groups (-OH) attached to a benzene ring, along with a carboxylic acid group (-COOH).

### 2.2. Plant Extraction

Fresh Ammi plants and Parsley samples were collected carefully and ground. The resulting plants were soaked in boiling water for 48 h. Subsequently, the infused water was carefully separated and subjected to freeze-drying to obtain a concentrated extract [13].

### 2.3. Calcium Oxalate Disintegration Study (Mimic Kidney Stones)

For each plant, varying amounts of the resultant ground from Ammi and Parsley were freeze-dried at different amounts 1, 2, 3, 4, and 5 g [13]. These quantities were then individually dissolved in 50 mL of distilled water to prepare different concentrations of plant extracts. Following the preparation of the extracts, oxalate crystals were selected as the test samples and immersed in the respective extract solutions. The samples were then allowed to soak for ours. Subsequently, the dimensions of the oxalate crystals were recorded for comparative analysis.

### 2.4. Total Phenolic Contents

**Figure 3:**
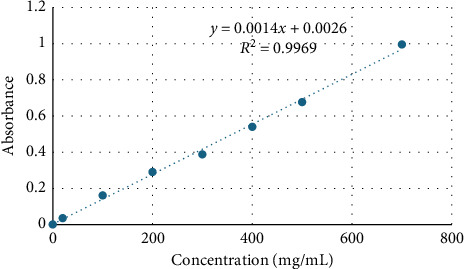
Standard curve of gallic acid.

To establish a standard gallic acid curve, dilutions ranging from 100 to 700 mg/mL were prepared in methanol from a stock solution of gallic acid. Each dilution (150 μL) was then mixed with 450 μL of water, followed by the addition of the Folin–Ciocalteu reagent (2.5 mL of Folin–Ciocalteu reagent). After a 5 min incubation period, 2 mL of 75 g/L sodium carbonate solution was added. The resulting solution was incubated with shaking at 30°C for 1.5 h. Subsequently, absorbance was measured at 765 nm using a spectrometer. The total phenolic content of the plants was quantified in terms of gallic acid equivalents (mg GAE/g). All experiments were conducted in triplicates to ensure accuracy.  Figure 3 illustrates the standard gallic acid curve and regression equation used to determine the determination of total phenolic content in the extracts [14].

### 2.5. Determination of the Total Phenolic Content

The total phenolic content was assessed using the Folin–Ciocalteu method. One consistent volume of the Folin–Ciocalteu reagent was used throughout the assay. Specifically, 150 μL of each plant extract was combined with 2.5 mL of 0.2 N Folin–Ciocalteu reagent and allowed to stand at room temperature for 5 min. Subsequently, 2 mL of a 75 g/L sodium bicarbonate solution was added, and the mixture was incubated at 30°C for 90 min. Absorbance readings were taken at 765 nm using a UV/Vis spectrophotometer (SPUV-26/TECH). Quantification of total phenolic content was performed by comparison with a gallic acid standard curve constructed from seven concentrations ranging from 100 to 700 μg/mL. This standard curve allowed interpolation of the results, which were expressed as milligrams of gallic acid equivalents (mg GAE) per gram of dried plant material. Concentrations were expressed as milligrams of gallic acid equivalents (mg GAE/g) of the dry extract [15]. The total phenolic content was evaluated for five different concentrations (1, 2, 3, 4, and 5 g/50 mL) of the freeze-dried powder of Ammi and Parsley plants. The total phenolic content was evaluated for five different concentrations from each plant after soaking for 24 h in water, as shown in Table 1.

### 2.6. DPPH Radical Scavenging Assay

The antioxidant properties of the extracts were evaluated using a 2,2-diphenyl-1-picrylhydrazyl (DPPH) free radical scavenging assay. To ensure the stability of the DPPH reagent, a 0.5 mM solution was freshly prepared in 100% methanol. The solution was mixed thoroughly and allowed to stabilize at room temperature for 30 min prior to use. During this stabilization period, the absorbance at 517 nm was measured periodically to confirm that the baseline absorption remained constant. Only when the absorbance demonstrated stability was the reagent used in subsequent assays. This step was performed to ensure reproducibility and reliability in the evaluation of antioxidant activity. Each experimental run included freshly prepared DPPH solution to maintain the accuracy of the results. In this method, the oxidized form of DPPH imparts a deep violet color to the methanol. Antioxidant compounds reduce DPPH by donating an electron, leading to a color change from violet to yellow. The absorbance of a freshly prepared 0.5 mM DPPH solution in 100% methanol was measured at 517 nm. Subsequently, 2 mL of the DPPH solution was added to 150 μL of the pure extract. The samples were measured twice: initially at zero time and then after 30 min of incubation in the dark (all measurements were conducted at 517 nm). Each sample was measured three times and the average readings were calculated. The antioxidant activity was evaluated for five different concentrations (1, 2, 3, 4, and 5 g/50 mL) of freeze-dried powder of Ammi and Parsley plants. The total phenolic content was evaluated for five different concentrations from each plant after soaking for 24 h in water, as shown in Table 1. The following equation was used to determine the percentage of DPPH inhibition by the extract [16]:

(1) $$\begin{array}{c} \%\text{ Inhibition}=\frac{\left(A-B\right)}{A}*100, \end{array}$$

where A is the absorbance of DPPH after the addition of extract at time zero and B is the absorbance of a sample taken after 30 min of reaction with DPPH [2].

The %inhibition/100 mg was calculated using the following equation:

(2) $$\begin{array}{c} \%\text{ inhibition}/100\text{mg}=\frac{\left(weight\;of\;the\;extracted\;sample*100\right)}{\left(\%\text{ inhibition}\right)}. \end{array}$$

### 2.7. Dissolution Study

#### 2.7.1. The Preparation of Simulated Gastric Fluid and Simulated Intestinal Fluid

The in vitro dissolution profiles of Ammi and Parsley extracts were investigated using simulated gastric fluid (SGF) and simulated intestinal fluid (SIF). Both media were prepared according to established protocols [17].

SGF: SGF was prepared by dissolving 2.0 g of sodium chloride (NaCl) and 3.2 g of pepsin in 1 L of distilled water. The pH was adjusted to 1.2 using hydrochloric acid (HCl) to mimic the acidic environment of the stomach. Pepsin was included to replicate the enzymatic activity present in gastric conditions.

SIF: SIF was prepared by dissolving 6.8 g of potassium dihydrogen phosphate (KH_2_PO_4_) in 250 mL of distilled water, followed by the addition of 77 mL of 0.2 M sodium hydroxide (NaOH) and sufficient distilled water to make 1 L of solution. The pH was adjusted to 6.8, replicating the slightly alkaline conditions of the small intestine.

Each medium was freshly prepared prior to the dissolution experiments and maintained at a constant temperature of 37°C throughout the study. The dissolution tests were conducted using a USP Type II paddle apparatus with a rotation speed of 100 rpm. Aliquots were withdrawn at predetermined time intervals and replaced with fresh medium to maintain sink conditions. These aliquots were filtered through a 0.45 μm membrane before analysis.

The in vitro dissolution profiles of Ammi and Parsley extracts for phenolic content and antioxidant activity were investigated. The cumulative release percentage of the total phenolic content and antioxidant activity was assessed over time. Dissolution studies were carried out using a USP apparatus type 2, also known as the paddle method, at a constant temperature of 37°C. Stirring was achieved by using a rotating paddle set at a speed of 100 rpm. Each formulation consisted of 100 mg of the extract added directly to 900 mL of SGF devoid of pepsin. At predetermined intervals of 10, 15, 20, 25, 30, 35, 40, 45, 50, 55, and 60 min, aliquots were withdrawn and replaced with fresh SGF to maintain sink conditions throughout the 1 h gastric digestion simulation. Subsequently, the samples were filtered through a 0.45 μm membrane before UV analysis. Similarly, dissolution tests were conducted using SIF to mimic small intestinal conditions over a duration of 1 h. All dissolution experiments were performed in triplicates for each extract formulation. Discrepancies in the dissolution profiles were analyzed to gauge the potential influence of the sample on the bioaccessibility and delivery of Ammi and Parsley extracts under simulated digestive conditions [2, 18]. An in vitro dissolution study was conducted using extracted samples obtained from 5 g of ground powder of both Ammi and Parsley.

## 3. Results

### 3.1. Total Phenolic Content

**Figure 4:**
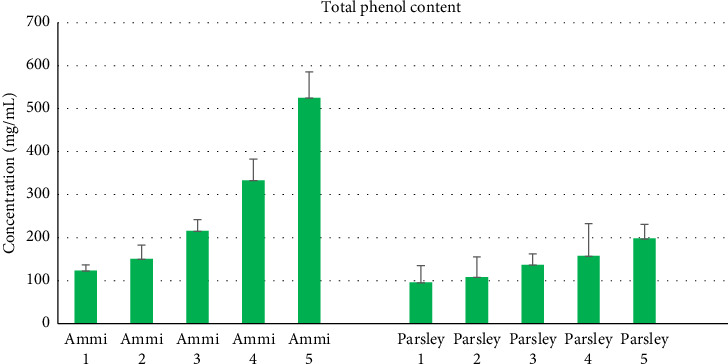
Total phenolic content for five different amounts of Ammi and Parsley.

The evaluation of total phenolic content was evaluated for varying quantities (1, 2, 3, 4, and 5 g/50 mL), respectively, of both Ammi and Parsley plants. It is evident that with an increase in the amount of extract, there was a corresponding increase in the total phenolic content, indicating a substantial presence of phenolic compounds in both plants (Figure 4). Moreover, it is noteworthy that Ammi exhibits a higher total phenolic content compared with Parsley across the tested concentrations [6, 19].

### 3.2. Antioxidant Activity

**Figure 5:**
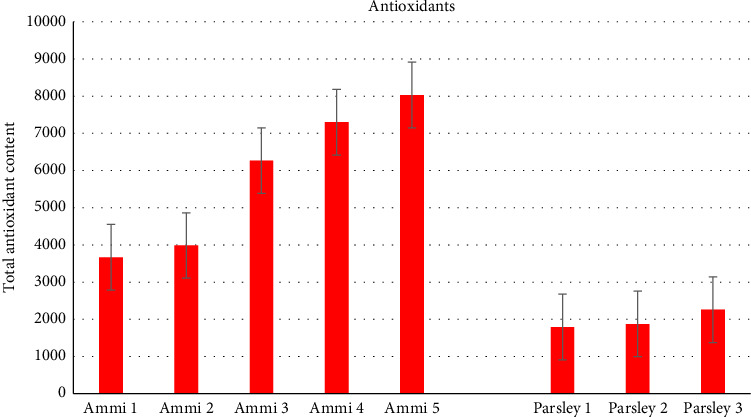
Antioxidant activity of five different amounts of Ammi and Parsley.

The antioxidant results indicated that, across all samples, Ammi demonstrated significantly higher antioxidant activity than Parsley. Moreover, there appeared to be a trend of increasing antioxidant activity with higher concentrations of both Ammi and Parsley extracts [19], as shown in Figure 5.

### 3.3. Calcium Oxalate Disintegration Study

**Figure 6:**
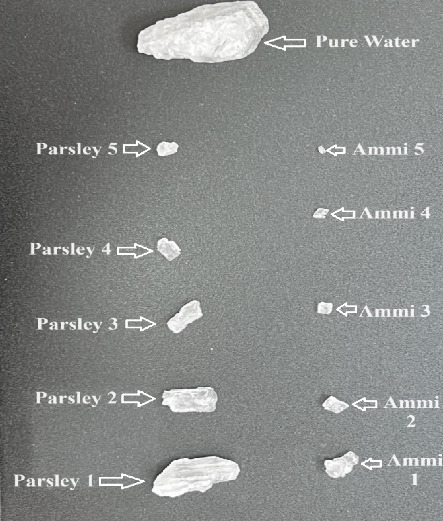
Calcium oxalate stones after soaking in different concentrations of Ammi, Parsley extract, and pure water as controls.

After soaking the Ammi and Parsley in water, the resulting water was freeze-dried and the resultant powder was dissolved in water at five different concentrations, as described in Section 2.3. Approximately five calcium oxalate stones were soaked in five different concentrations of Ammi and Parsley extracts for 24 h. Calcium oxalate stones were chosen because they are one of the most stubborn types of kidney stones [20]. The size of these stones was evaluated using a micrometer screw gauge to measure their width after 24 h of soaking in plant extracts [21, 22]. Five similar-sized calcium oxalate stones were chosen and soaked separately with each plant extract. Therefore, we assessed the disintegration ability of Ammi and Parsley extracts. For control reasons, oxalate stones were soaked in pure water [12]. The results shown in Figure 6 reveal a significant decrease in the size of oxalate stones in both plant extracts. However, the reduction in stone size was notably greater in the Ammi extract than in the Parsley extract and pure water samples.

**Figure 7:**
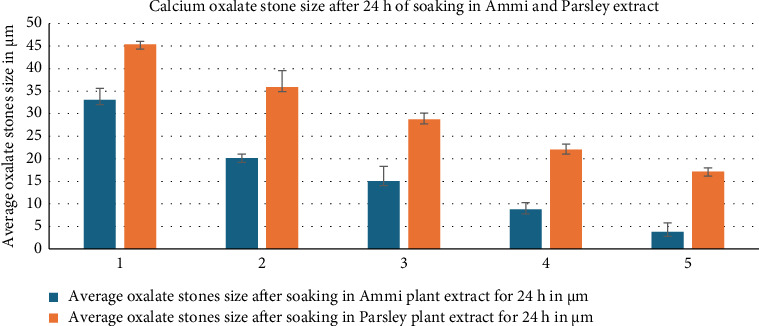
Average calcium oxalate stone sizes after soaking in Ammi, Parsley, and pure water for 24 h.

After 24 h of soaking oxalate stones in various concentrations of Ammi and Parsley plant extracts, significant reductions in average stone size were observed compared with the control group immersed in water alone (Figure 7). In the case of Ammi plant extract, as the concentration increased from 1 g/50 mL to 5 g/50 mL, a notable decrease in average stone size was evident, ranging from 33.05 to 3.79 μm, respectively. Similarly, with the Parsley plant extract, there was a consistent reduction in average stone size with increasing concentrations, ranging from 45.36 μm at 1 g/50 mL to 17.14 μm at 5 g/50 mL. However, it is noteworthy that the effect appeared to be more pronounced with Ammi extract than with Parsley extract, indicating a stronger therapeutic potential of Ammi in reducing oxalate stone size. These findings suggest a dose-dependent effect of both Ammi and Parsley extracts in reducing the size of oxalate stones after 24 h of soaking. Compared with the control group, with an average stone size of 921.33 μm, the experimental groups treated with plant extracts exhibited remarkable reductions in stone size, underscoring the potential therapeutic efficacy of Ammi and Parsley in managing oxalate stone formation, with a more significant impact observed with the Ammi extract [13].

### 3.4. Dissolution Study

**Figure 8:**
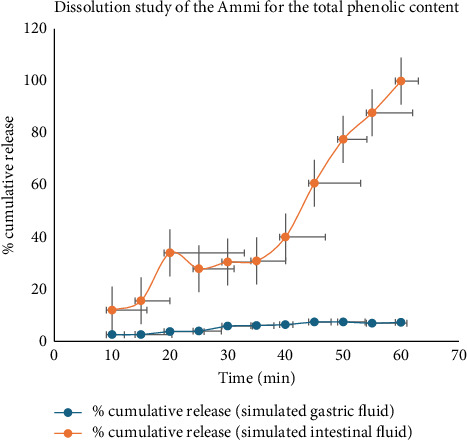
Dissolution profile of the total phenolic content of Ammi plant extracts.

The dissolution profile (Figure 8) of the total phenolic content from Ammi extract was investigated in simulated gastric and intestinal fluid media, providing valuable insights into its release behavior over time. In SGF, there was a gradual increase in the cumulative release of phenolic content from the Ammi extract throughout the duration of the test. Notably, at the 60 min mark, the cumulative release reached 7.33%, indicating a sustained liberation of phenolic compounds under simulated conditions. Conversely, in the SIF, a similar trend of increasing cumulative release was observed, with a more pronounced release of phenolic content compared with gastric conditions. At the 60 min mark, the cumulative release reached 100%, suggesting complete dissolution and substantial liberation of phenolic compounds under intestinal-simulated conditions. It is noteworthy that variations in cumulative release were observed between the gastric and intestinal media, reflecting differences in the dissolution behavior of phenolic compounds from the Ammi extract under varying physiological conditions. These findings contribute to a better understanding of the potential bioavailability and absorption kinetics of phenolic compounds from Ammi extracts in the gastrointestinal tract [12, 22].

**Figure 9:**
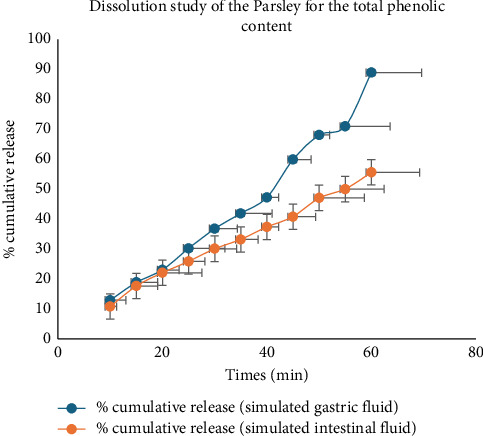
Dissolution profile of the total phenolic content of Parsley plant extract.

The analysis of total phenolic content from Parsley extract in simulated gastric and intestinal fluid media revealed distinctive dissolution patterns over time (Figure 9). In the SGF, there was a progressive increase in the cumulative release of phenolic content from the Parsley extract throughout the experimental period. By the end of the 60 min period, the cumulative release reached 88.92%, indicating substantial liberation of phenolic compounds under gastric-simulated conditions. Conversely, in SIF, a similar trend of increasing cumulative release was observed, with notable differences compared with gastric conditions. At the 60 min mark, the cumulative release reached 55.6%, reflecting the continued release and bioavailability of phenolic compounds under intestinal-simulated conditions. It is noteworthy that variations in cumulative release were observed between gastric and intestinal media, underscoring differences in the dissolution behavior of phenolic compounds from Parsley extracts under distinct physiological conditions. These findings contribute to a deeper understanding of the potential bioavailability and absorption kinetics of phenolic compounds from Parsley extracts in the gastrointestinal tract.

**Figure 10:**
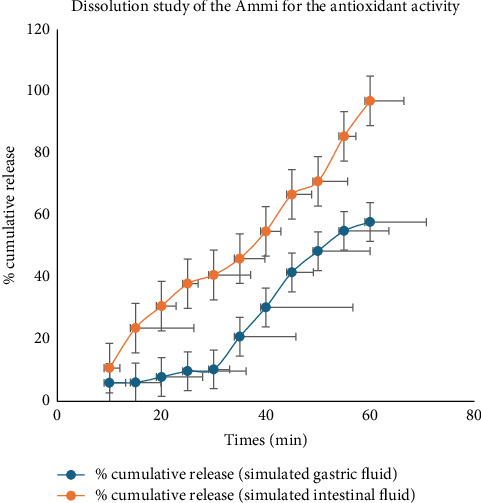
Dissolution profile of the antioxidant activity of Ammi plant extracts.

The assessment of antioxidant activity of Ammi extract through a dissolution test in simulated gastric and intestinal fluid media revealed distinctive release patterns over time (Figure 10). In SGF, there was a gradual increase in the cumulative release of antioxidant activity from the Ammi extract throughout the experimental period. By the end of the 60 min period, the cumulative release reached 57.89%, indicating a significant liberation of antioxidant compounds under gastric-simulated conditions. Conversely, in SIF, a similar trend of increasing cumulative release was observed, with notable differences compared with gastric conditions. At the 60 min mark, the cumulative release reached 97.01%, reflecting the continued release and bioavailability of antioxidant compounds in intestinal-simulated conditions. It is noteworthy that variations in cumulative release were observed between the gastric and intestinal media, highlighting differences in the dissolution behavior of antioxidant compounds from Ammi extract under distinct physiological conditions. These findings contribute to a deeper understanding of the potential bioavailability and absorption kinetics of antioxidant compounds from Ammi extracts within the gastrointestinal tract.

**Figure 11:**
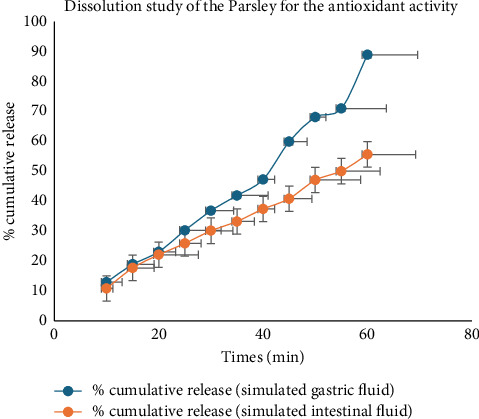
Dissolution profile of the antioxidant activity of Parsley plant extracts.

The evaluation of antioxidant activity of Parsley extract via a dissolution test in simulated gastric and intestinal fluid media revealed distinct release profiles over the course of the experiment (Figure 11). In SGF, there was a steady increase in the cumulative release of antioxidant activity from the Parsley extract throughout the testing period. From the conclusion of the 60 min duration, the cumulative release reached 89.44%, indicating significant liberation of antioxidant compounds under gastric-simulated conditions. Conversely, in SIF, a similar trend of increasing cumulative release was observed, albeit with notable differences compared with gastric conditions. At the 60 min mark, the cumulative release reached 69.26%, reflecting the continued release and bioavailability of antioxidant compounds under intestinal-simulated conditions. Notably, variations in cumulative release were observed between gastric and intestinal media, suggesting differences in the dissolution behavior of antioxidant compounds from Parsley extracts under distinct physiological conditions. These findings contribute to a better understanding of the potential bioavailability and absorption kinetics of antioxidant compounds from Parsley extracts in the gastrointestinal tract.

## 4. Discussion

This study investigated the therapeutic potential of *Ammi visnaga* (Ammi) and *Petroselinum crispum* (Parsley) extracts in terms of their antioxidant activity, total phenolic content, and efficacy in disintegrating calcium oxalate kidney stones. These plants have been traditionally recognized for their pharmacological properties, particularly their antioxidant potential, which is attributed to the presence of phenolic compounds [23]. This study aimed to provide comprehensive insights into the pharmaceutical properties of these botanical extracts, including their dissolution behavior, antioxidant activity, and phenolic content, to evaluate their therapeutic relevance. The assessment of antioxidant activity and total phenolic content revealed concentration-dependent increases in both parameters for the Ammi and Parsley extracts. This indicates the presence of potent antioxidants in these plants that play a crucial role in scavenging free radicals and mitigating oxidative stress-related damage in the body. The higher antioxidant activity observed in Ammi than in Parsley suggests that Ammi extract may possess stronger antioxidant properties, potentially due to variations in phenolic composition or concentration between the two plants [24]. This study demonstrated the efficacy of Ammi and Parsley extracts in disintegrating calcium oxalate kidney stones, a common urinary tract ailment. The significant reduction in stone size observed after soaking oxalate stones in both plant extracts highlights their potential therapeutic utility for managing kidney stone formation. Notably, the Ammi extract exhibited a more pronounced effect in reducing stone size than the Parsley extract, indicating its superior efficacy in disintegrating oxalate stones. This finding underscores the importance of further exploring the mechanisms underlying the stone-disintegrating properties of these botanical extracts and their potential clinical application in managing kidney stone-related disorders. The dissolution study provided valuable insights into the release behavior of phenolic content and antioxidant activity of Ammi and Parsley extracts in simulated gastric and intestinal fluid media. The results revealed distinct release profiles for both parameters, with variations observed between gastric and intestinal conditions. Notably, the Ammi extract exhibited sustained release of phenolic content and antioxidant activity in both gastric and intestinal media, indicating its potential for prolonged therapeutic action. Similarly, the Parsley extract demonstrated substantial release of phenolic compounds and antioxidant activity, albeit with differences in release kinetics compared with the Ammi extract [25, 26]. These findings suggest that Ammi and Parsley extracts may exhibit different dissolution behaviors, potentially influencing their bioavailability and therapeutic efficacy in vivo.

## 5. Conclusion

This study highlights the therapeutic potential of *Ammi visnaga* and *Petroselinum crispum* extracts due to their significant antioxidant activity, high phenolic content, and ability to disintegrate calcium oxalate kidney stones. Both extracts demonstrated dose-dependent efficacy, with Ammi showing superior antioxidant and stone-disintegrating properties compared with Parsley. These findings suggest that Ammi and Parsley extracts could be promising candidates for managing oxidative stress-related conditions and kidney stone formation. Further research is recommended to elucidate their mechanisms of action and optimize formulations for clinical applications.

## Figures and Tables

**Table 1:** Samples abbreviation.

Amount of the sample from Ammi and Parsley in grams/50 mL water	Ammi samples names	Parsley samples name
1	Ammi 1	Parsley 1
2	Ammi 2	Parsley 2
3	Ammi 3	Parsley 3
4	Ammi 4	Parsley 4
5	Ammi 5	Parsley 5

## Data Availability

The data that support the findings of this study are available from the corresponding author upon reasonable request.
